# Supplementary material for: Extra-embryonic tissue spreading directs early embryo morphogenesis in killifish
Source: Nat Commun. 2017 Jun 5;8:15431. doi: 10.1038/ncomms15431 (PMC5465322; doi:10.1038/ncomms15431)
Supplement: Supplementary Information — Supplementary Figures, Supplementary Table, Supplementary Reference. [file ncomms15431-s1.pdf]

Supplementary Figures

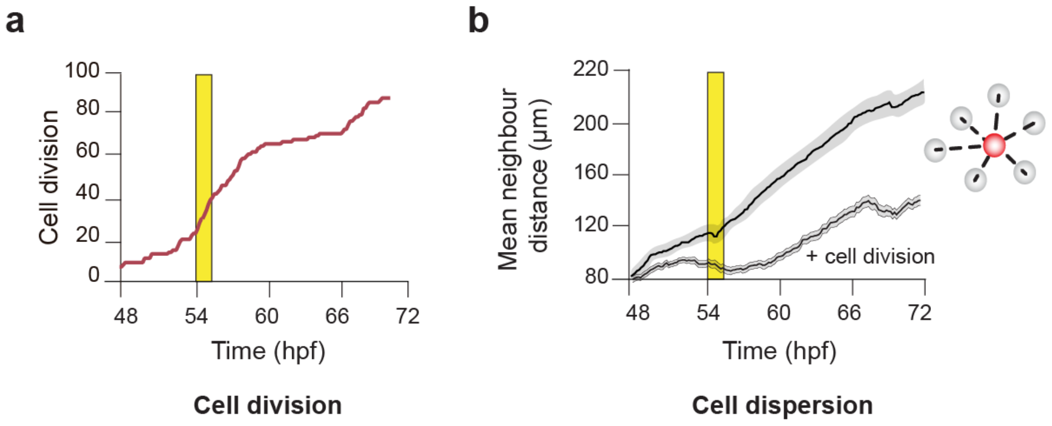

Supplementary Figure 1 | Dynamics of DCL cell division and its relation to cell dispersion during epiboly of *A. nigripinnis*. (**a**) Temporal changes in the cumulative number of DCL cell divisions between late blastula (48 hpf) and 60 % epiboly (72 hpf). Two main phases of increased cell divisions are detected between 54-60 hpf (early epiboly) and after 68 hpf (mid epiboly). During the period of analysis, virtually all cells of the DCL undergo one round of division. (**b**) Temporal changes in the mean distance to the six nearest neighbours within the DCL. During epiboly, DCL cells disperse as revealed by the steady increase in mean neighbour distance (top curve). As new neighbours appear after events of cell division and occupy available free spaces, the overall mean neighbour distance decreases (bottom curve). The vertical yellow line in **a** and **b** indicates the onset of epiboly, defined by the initial vegetal-ward movement of the EVL epithelial margin.

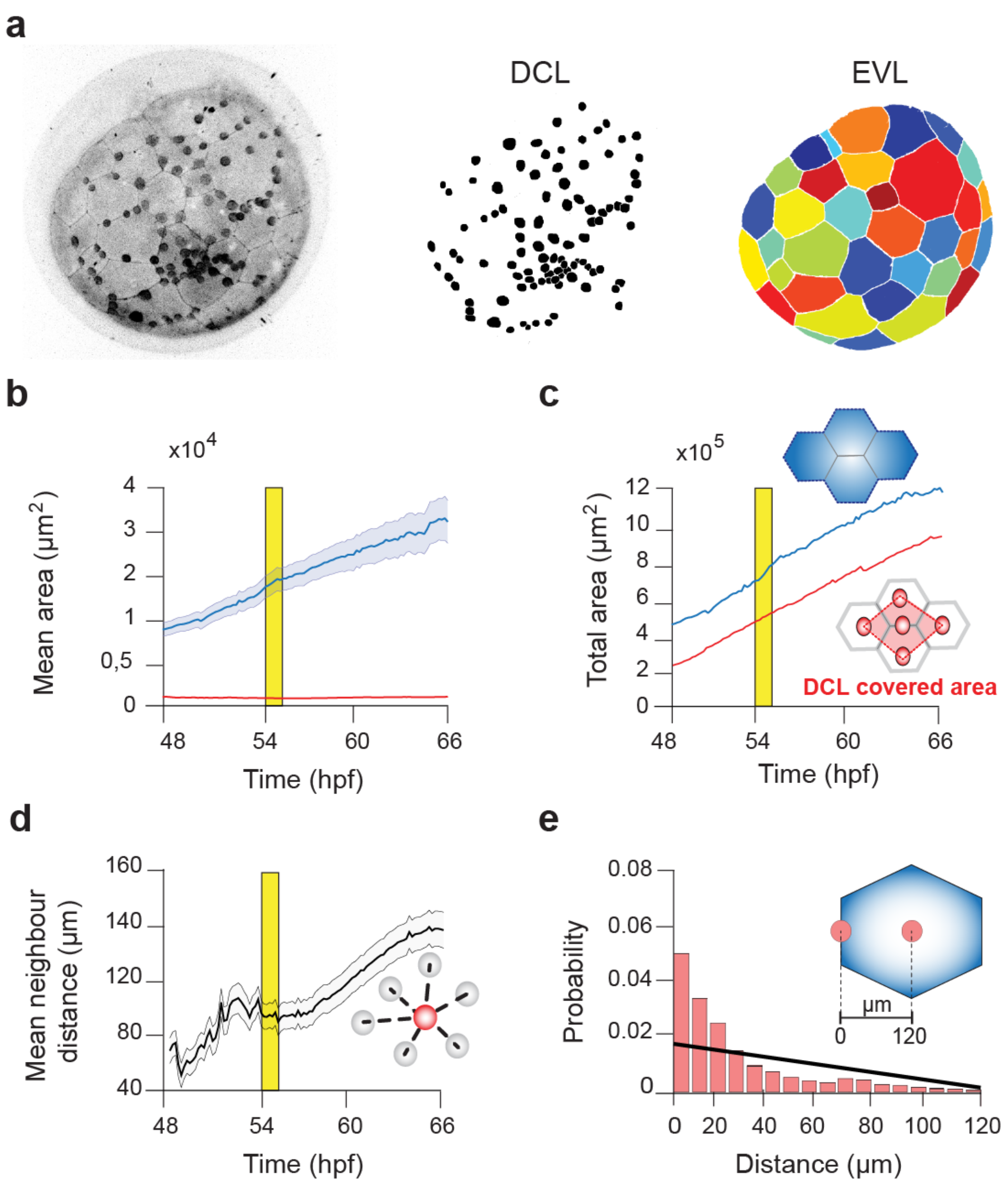

Supplementary Figure 2 | Dynamics of tissue spreading of the embryonic DCL and its relation to the surface expansion of the extra-embryonic EVL in annual killifish (replica). (**a**) An embryo of *A. nigripinnis* expressing *lifeact-GFP* was imaged from the animal pole by 4D confocal microscopy between late blastula (48 hpf) and 40 % epiboly (66 hpf) (left), and cells of the DCL (middle) and EVL (right) were segmented. Individual EVL cells are represented with different colours. (**b**) Temporal changes in the mean surface area of EVL and DCL cells, with values expressed as means ± SEM. (**c**) Temporal changes in the total area covered by the EVL and the convex area covered by the underlying DCL cells, measured as indicated in the top and bottom insets, respectively. (**d**) Temporal changes in the mean distance to the six nearest DCL neighbours, with values expressed as means ± SEM. (**e**) Probability distribution of DCL cell position as a function of the distance to EVL cell borders, for the period between 48-66 hpf. The black straight line corresponds to the expected random distribution for a mean EVL cell surface radius of 120 µm, as indicated in the top right corner. In all panels, red and blue colours correspond to the DCL and EVL, respectively. The vertical yellow line in b-d indicates the onset of epiboly, defined by the initial vegetal-ward movement of the EVL epithelial margin.

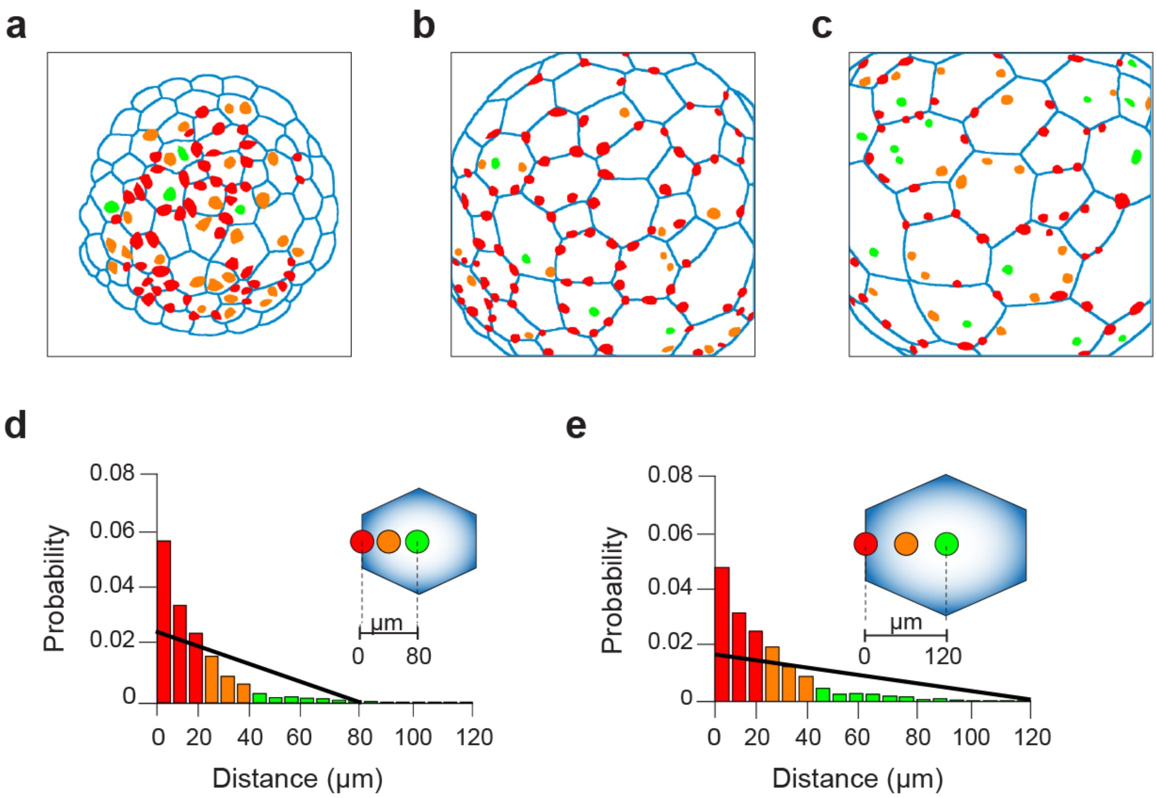

Supplementary Figure 3 | Spatial distribution of DCL cells with respect to EVL cell borders during early- and mid- epiboly stages in *A. nigripinnis* (related to Fig. 2e) (**a-c**) Animal pole views of embryos at late blastula (a, 48 hpf), 40 % epiboly (b, 64 hpf) and 60 % epiboly (c, 64 hpf) showing the position of DCL cells (red, orange and green circles) with respect to EVL cell borders (blue lines). (**d**,**e**) Probability distributions of DCL cell position as a function of the distance to EVL cell borders for the period between 54-64 hpf (**d,** onset-40 % epiboly) and 64-72 hpf (**e**, 40-60 % epiboly). Red, orange and green colours indicate different distance intervals to EVL cell borders, as depicted in the schematics and plots of **d**,**e**.

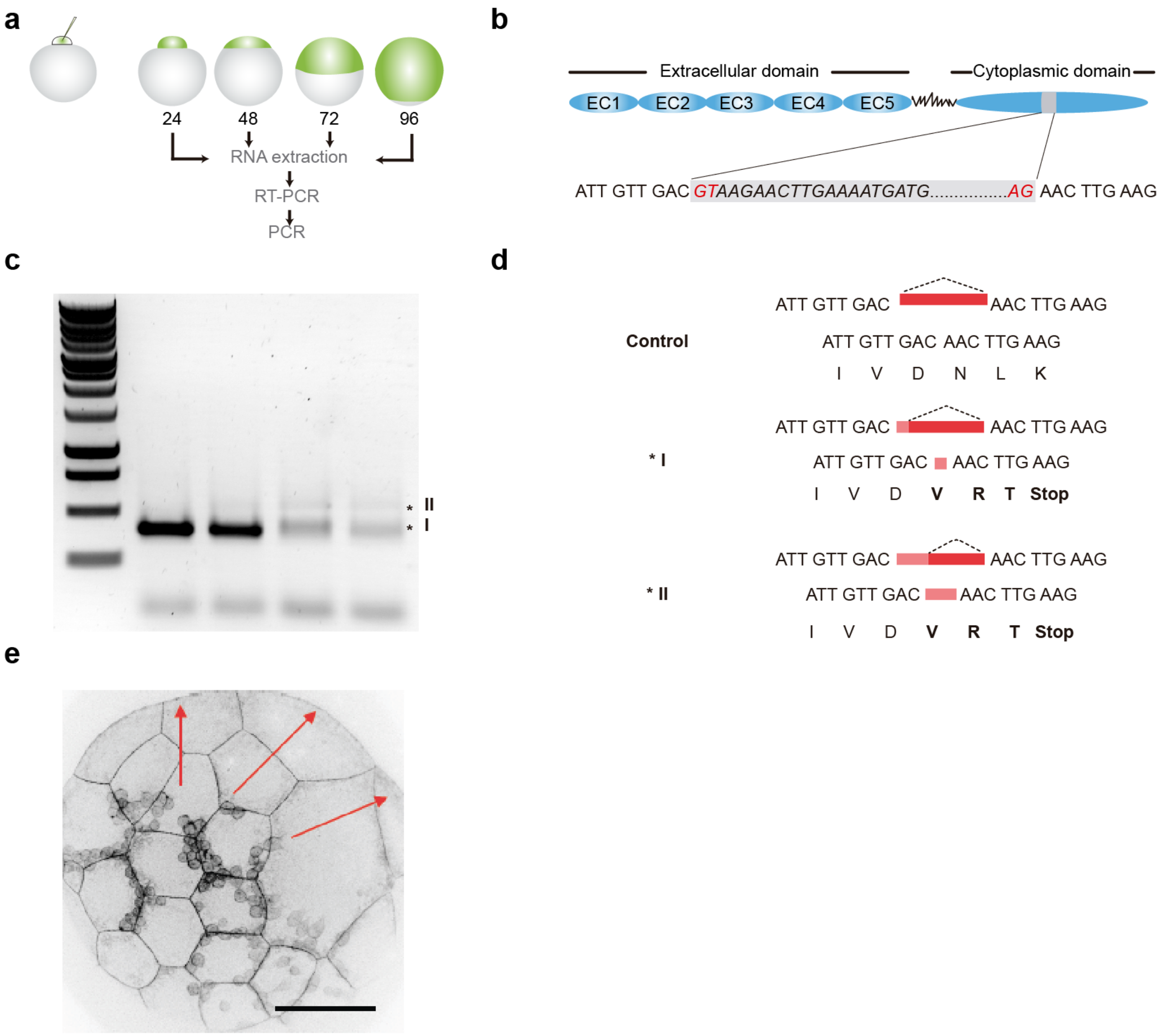

Supplementary Figure 4 | Knock down of *A. nigripinnis* E-cad using antisense morpholino oligonucleotides (related to Fig. 3d). (**a-d**) Steps for assessing the efficiency of *e-cad* morpholino knock down in *A. nigripinnis* (see details in Methods). (**a**) RNA was extracted from *e-cad* morpholino injected embryos for PCR amplification. (**b**) Schematic representation of the exon-intron boundary region within the intracellular domain of E-cad (shaded grey), with donor and acceptor splicing sites denoted with red letters. The splicing sequence targeted by morpholino is highlighted. (**c**) RT-PCR using a set of primers flanking the recognition site of the *e-cad* morpholino. A single PCR band seen at 24 hpf is progressively converted into two higher weight bands from 48 to 96 hpf (* I and II). (**d**) Sequencing of the higher weight bands shown in **c** reveal the presence of residual intron sequence (pink) in the mature mRNA, confirming the efficiency of morpholino targeting. (**e**) Animal pole view of a 50 % epiboly embryo after injection of *e-cad* morpholino at 1 cell stage. Global E-cad knock down induces a delay of DCL epiboly with respect to the normal epibolic expansion of the EVL margin (arrows). Images correspond to confocal microcopy z-stack maximum projections, with an inverted look-up table. Scale bar, 250 μm.

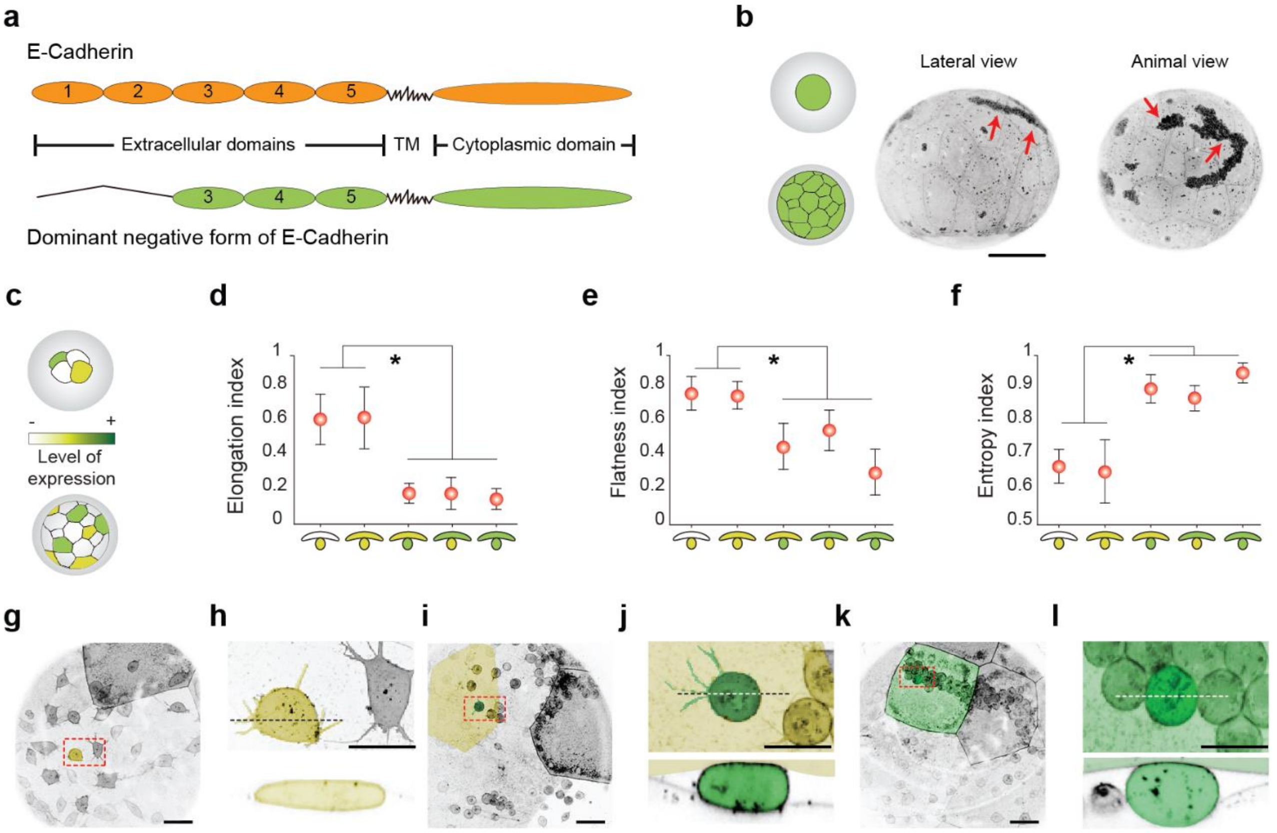

Supplementary Figure 5 | Functional abrogation of E-cadherin in *A. nigripinnis* using a dominant negative strategy (related to Fig. 3c-h). (**a**) Schematic representation of wild type E-cadherin (orange) and dominant negative form of E-cad (dn E-cad, green). (**b**) Lateral and animal pole views of a 90 % epiboly embryo after global over-expression of *dn e-cad*. DCL spreading is severely disrupted, with cells forming clusters at the animal pole (red arrows). (**c-l**) Assessment of the changes in DCL cell shape after mosaic expression of *dn e-cad* in a subset of cells of the DCL, EVL or both. (**c**) Schematic diagram of the experimental strategy based on the co-injection of variable amounts of *dn e-cad* + *GAP43-EGFP* mRNA mix in single blastomeres at 4-cell stage. The colour scale indicates the strength of *dn e-cad* expression. (**d-f**) Graphs showing the values of three DCL cell shape parameters in the different experimental conditions depicted below the x-axis. For each cell, the three principal axes were computed from the segmented image using 2nd order moments [1] (see Methods). In the graphs, for each condition, circles and banana-like icons represent DCL and EVL cells, respectively, with colours indicating the strength of *dn e-cad* expression, as in **c**. DCL cells become rounded when E-cad function is severely abrogated in the DCL, EVL, or in both cell types, as indicated by the decrease in elongation (**d**) and flatness (**e**), and increase in entropy index (**f**). (**g-l**) Examples of DCL cell shape phenotypes from a subset of conditions shown in **d-f**, as revealed in whole embryo views (**g**,**i**,**k**) and in high magnification superficial (top panels in **h**,**j**,**l**) and orthogonal (bottom panels in **h**,**j**,**l**) views of DCL cells. Images in **g-l** (as in **b**) correspond to confocal microcopy z-stack maximum projections, with an inverted look-up table. Scale bars, 30 μm.

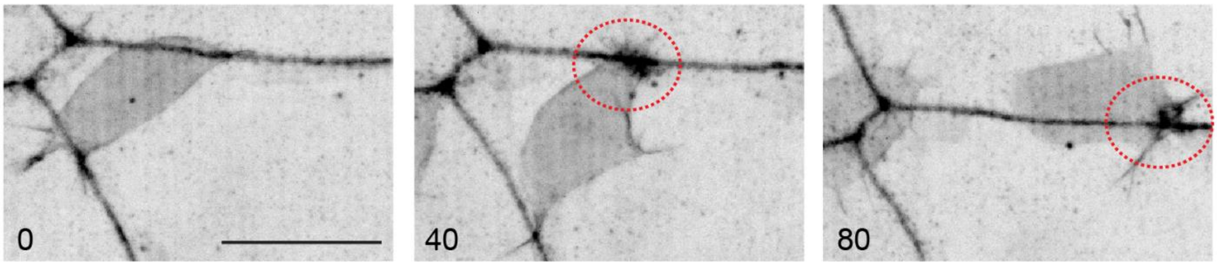

Supplementary Figure 6 | DCL cells sense EVL cell borders during epiboly of *A. nigripinnis*. Time lapse series of confocal microscopy z-stacks maximum projections of embryos expressing *lifeact-GFP*, revealing the presence of actin brushes (red circles) at transient contacts between DCL cells and the EVL cell border. Numbers at bottom-left corners of panels correspond to minutes. The look-up table has been inverted. Scale bar, 30 µm.

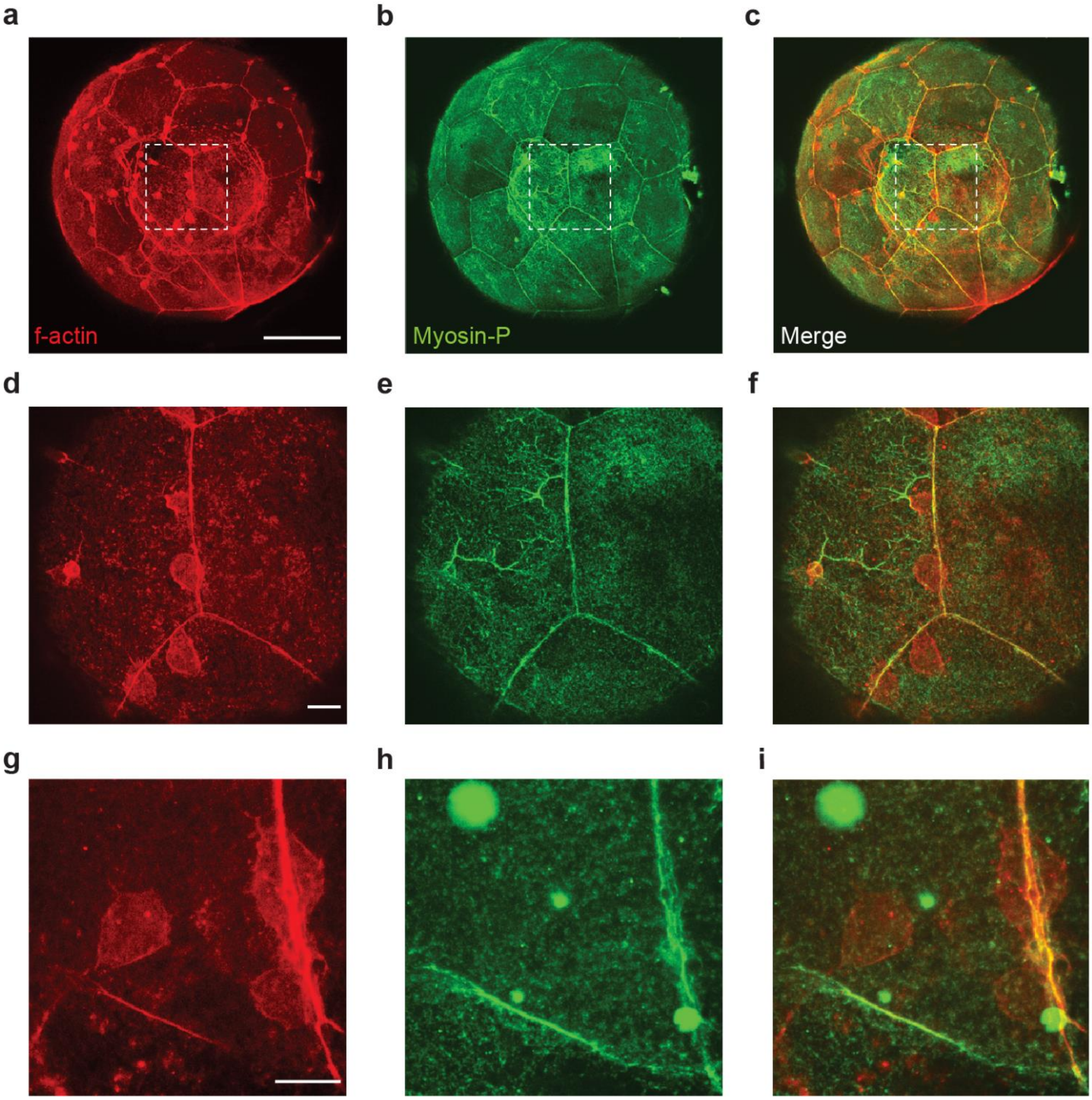

Supplementary Figure 7 | Phosphorylated non-muscular myosin II localises at EVL cell borders during epiboly of *A. nigripinnis*. Confocal z-stacks maximum projections of embryos at 60 % epiboly (72 hpf) after phalloidin staining to label F-actin (**a**,**d**,**g**), indirect immunofluorescence against phosphorylated non-muscular myosin II (**b**,**e**,**h**), and merge images (**c**,**f**,**i**). The dashed square regions depicted in **a-c** are shown at higher magnification in **d-f**, respectively. High magnification views of **g-i** correspond to a different embryo. Scale bars, 250 µm (**a-c**) and 30 µm (**d-i**).

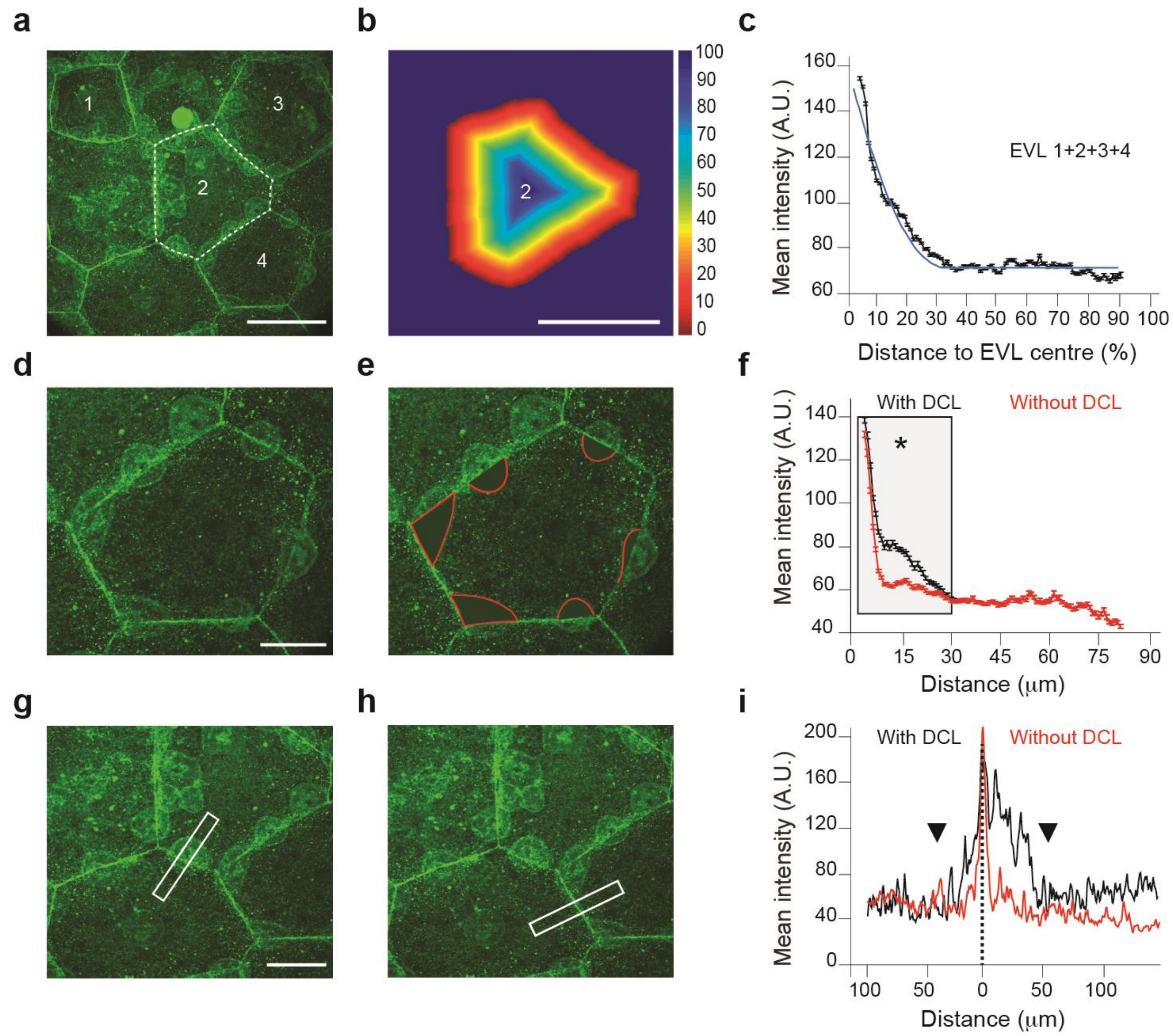

Supplementary Figure 8 | Quantification of fluorescence intensity reveals a lack of E-Cadherin protein gradient in EVL cells. (**a-c**) Quantification of E-cad signal relative to the distance to the EVL cell border. (**a**) Confocal z-stack maximum projection of a 50 % epiboly embryo after indirect immunofluorescence against E-cad. The 4 EVL cells used for analysis are indicated (1 to 4). (**b**) E-cad signal was measured at different distances to the EVL cell border (coloured contours). (**c**) Plot of the mean E-cad signal intensity at different distances from the EVL cell border. Distances are normalised to the total distance from the border to the centre (border=0 %, centre=100 %). (**d-f**) Analysis of E-cad fluorescent signal in a single EVL cell after excluding the fluorescent signal from DCL cells. (**d**) Confocal image of EVL cell 4. (**e**) Same as in **d** but excluding E-cad signal from all DCL cells located at the EVL cell border (outlined in red). Signal intensity in regions corresponding to DCL cells were replaced by background intensity. (**f**). Plot of E-cad signal intensity at different distances from the EVL cell border (distance=0) in the presence (black, corresponding to panel **d**) and absence (red, corresponding to panel **e**) of DCL cells. As observed in panel **c**, E-cad signal in the presence of DCL cells shows a gradual increase in the first 30 μm of the EVL cell border (highlighted box). This gradual increase is not observed when the fluorescence signal from DCL cells are excluded from the analysis, (boxed area) *$p<0.05$ (Wilkoxon). (**g-i**) Analysis of E-cad signal in regions of the EVL containing and devoid of DCL cells. (**g**) Confocal image depicting the band of E-cad signal quantification across the border of EVL cell 2, which contains a single DCL cell. (**h**) Confocal image depicting the band of E-cad signal quantification across the border of EVL cell 4, which is devoid of DCL

cells. (**i**) Plot of E-cad signal intensity for the regions of analysis depicted in **g** and **h**. The gradual increase in E-cad signal observed in the vicinity of the EVL cell border in the presence of a DCL cell (black line, starting at arrowheads) is not observed across the EVL cell border devoid of DCL cells (red line). Scale bars, 100 µm (**a**,**b**) and 50 µm (**d**,**e**,**g**,**h**)

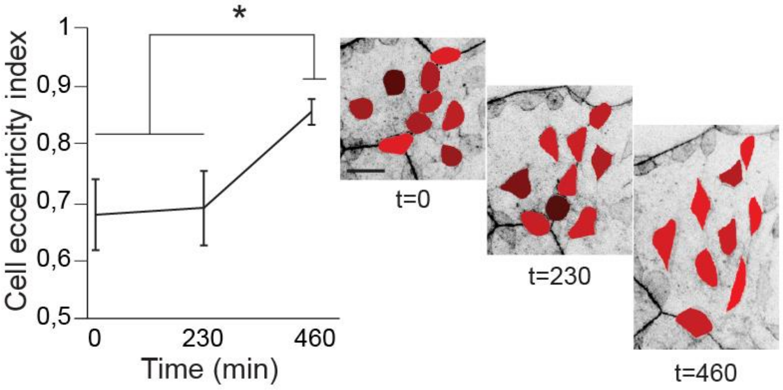

Supplementary Figure 9 | Changes in DCL cell shape as two EVL cells undergo fusion during epiboly of *A. nigripinnis*. Quantification of the temporal progression in DCL cell eccentricity (plot on the left) as two overlying EVL cells undergo fusion of their membranes (confocal microscopy temporal series on the right. DCL cells have been pseudo-coloured in red according to their value of eccentricity index, increasing from black to red). Values are expressed as means ± SEM. (n=9 cells) *p<0.05 (Wilkoxon) (from Supplementary Movie 8). Scale bar, 30 µm.

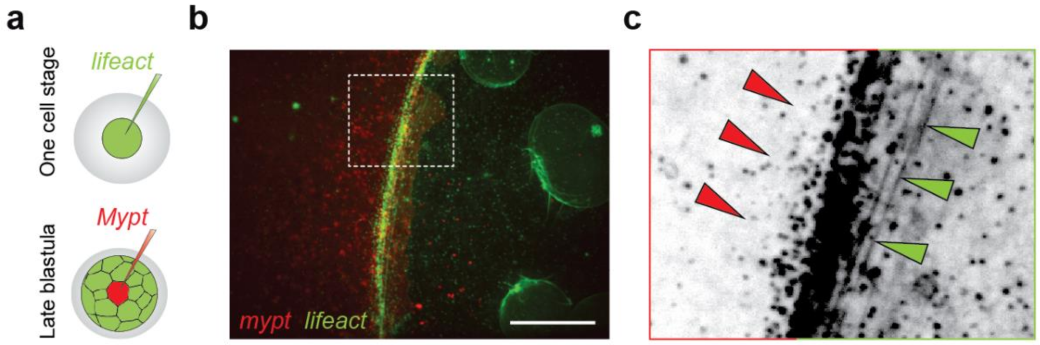

Supplementary Figure 10 | Effect of local over-expression of *N-ter-MYPT1* in a single EVL cell during epiboly of *A. nigripinnis*. (**a**) Schematic diagram of the two step mRNA injection protocol: *lifeact-GFP* is injected at 1-cell stage while *N-ter-MYPT1* (*mypt*) is injected in a single EVL cell together with rhodamine-dextran (tracer) at blastula stages. (**b**) Confocal microscopy z-stacks maximum projections of a 60 % epiboly embryo (72 hpf) expressing *N-ter-MYPT1* + rhodamine-dextran in a single EVL cell, showing the border between a *mypt* (+) (red) and a *mypt* (-) (green) EVL cell. (**c**) High magnification view of *lifeact-GFP* expression from the square region depicted in **b**, showing considerable reduction of cortical actin cables along the border of the *mypt* (+) EVL cell (red arrowheads) compared to the control *mypt* (-) EVL cell (green arrowheads). Scale bar, 30 µm.

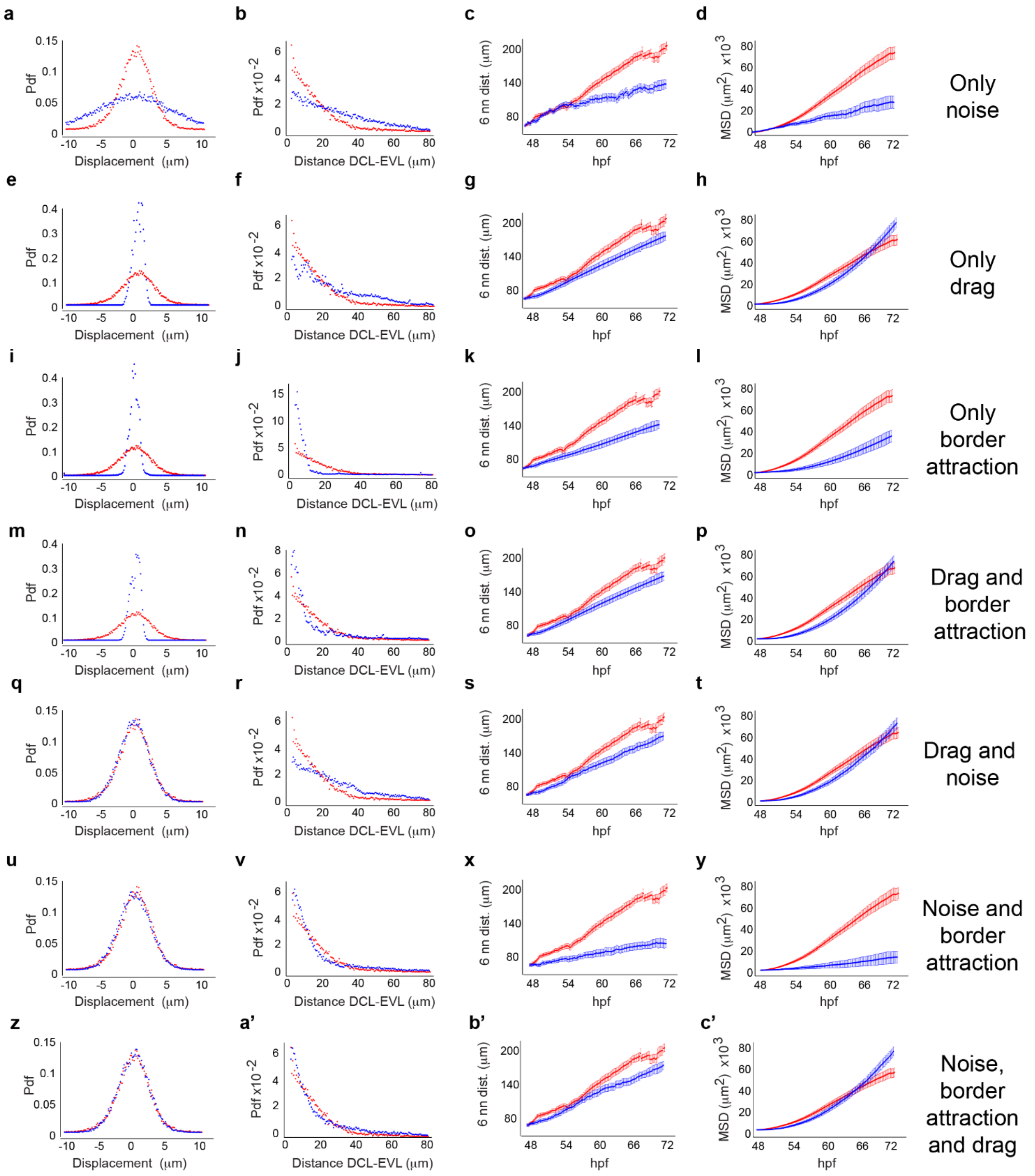

Supplementary Figure 11 | Comparison of the performance of simulations considering different combinations of forces. In all combinations, from left to right, simulations (blue) are compared to experiments (red) for the probability density function of the displacement of DCL cells (first column, left), the probability distribution of the distance of DCL cells to the nearest EVL cell border (second column), the mean distance to the six nearest neighbours within the DCL (third column), and the mean square displacement (fourth column). The different combination of forces are indicated on the right. Simulations considering only noise (**a-d**) and only border attraction (**i-l**) show the worst performance, deviating from experiments in all figures of merit. Combination of noise and border attraction (**u-y**) recapitulate DCL

displacement and distance to EVL cell border but fail to recapitulate measures of dispersion (MSD and neighbour distance) while the opposite is seen in the combination of drag and border attraction (**m-p**), and when considering only drag (**e-h**). Combination of drag and noise recapitulate almost all experimental conditions with the exception of distance to the EVL cell border (**q-t**). Finally, the best performance in all figures of merit is observed when considering noise, drag and EVL cell border attraction (**z-c'**).

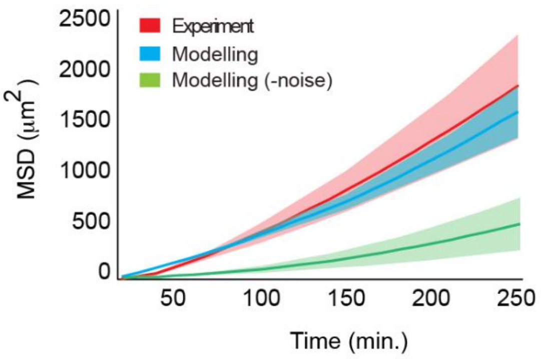

Supplementary Figure 12 | Removal of random walk from simulations reduce the Mean Square Displacement of DCL cells. The experiment (red) and simulation with all terms (blue; including EVL dragging, random walk, and EVL cell border attraction) both exhibit a similar super-diffusive behaviour. When random walk is removed from simulations (green) the slope of the Mean Square Displacement (MSD) is reduced. To compute MSD, 9 trajectories visible in all frames starting close to the animal pole were taken into account for both experiment and simulations.

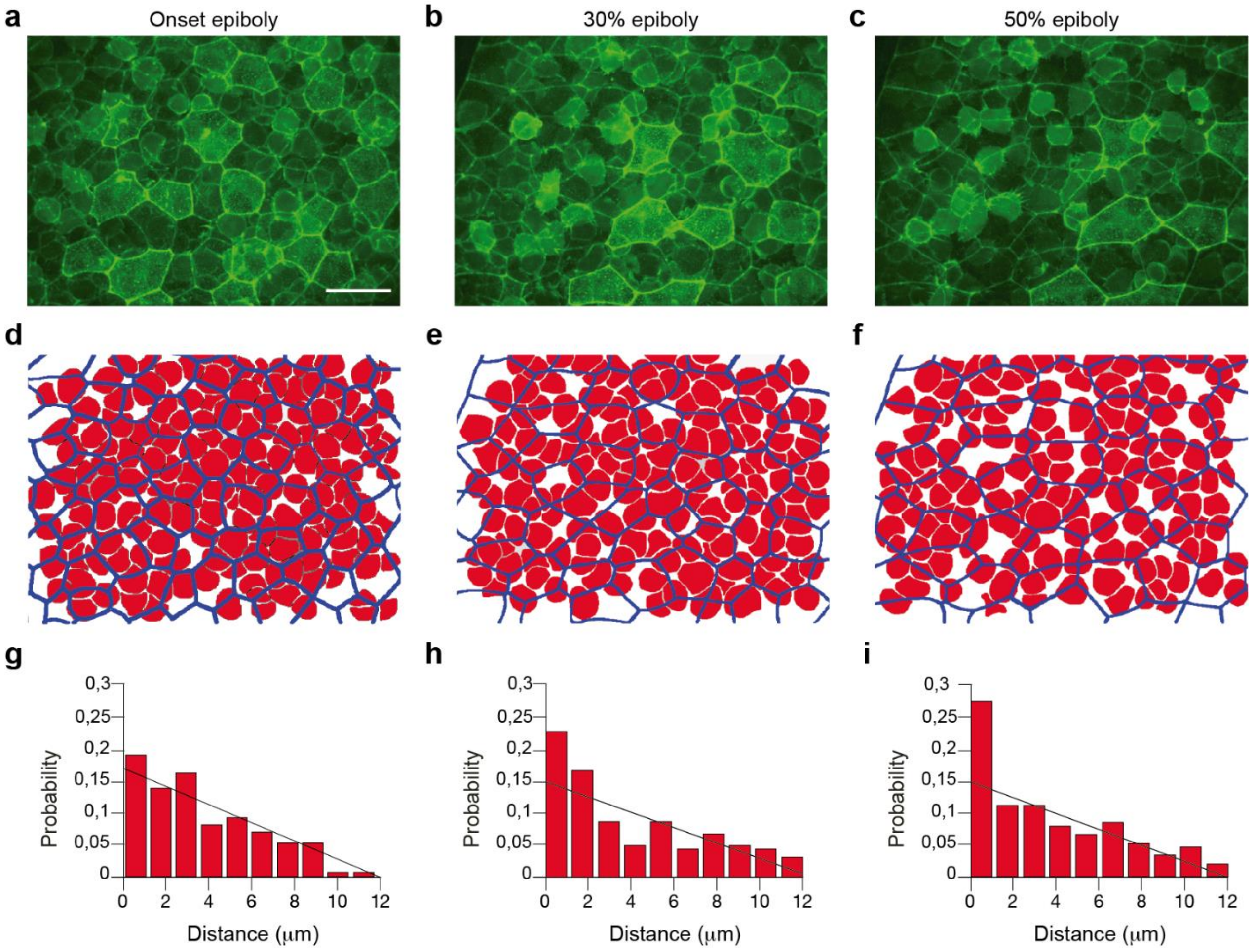

Supplementary Figure 13 | Spatial distribution of DCL cells with respect to EVL cell borders during epiboly in *Fundulopanchax gardnerii*. (**a-c**) Confocal microscopy z-stack maximum projections obtained at the animal pole of embryos expressing *lifeact-GFP* at the onset (**a**), 30 % (**b**) and 50 % (**c**) epiboly stages. (**d-f**) Representations of the segmented DCL (red dots) and EVL (blue lines) cells obtained from the images shown in **a-c**. (**g-i**) Probability distribution of DCL cell position as a function of the distance to EVL cell borders, as measured from the segmented images shown in **d-f**. The black straight line corresponds to the expected random distribution for a mean EVL cell surface radius of 50 µm. At the onset of epiboly, the probability distribution of DCL cells is close to random (**g**). However, as the EVL expands during epiboly and free space becomes available for DCL cells, they adopt a preferential distribution towards EVL cell borders (**h**,**i**). Comparison of histograms **g-i** using Kolmogorov-Smirnov (*$p<0.01$) revealed that **i** is significantly different to **h** and **g**. Scale bar, 100 µm.

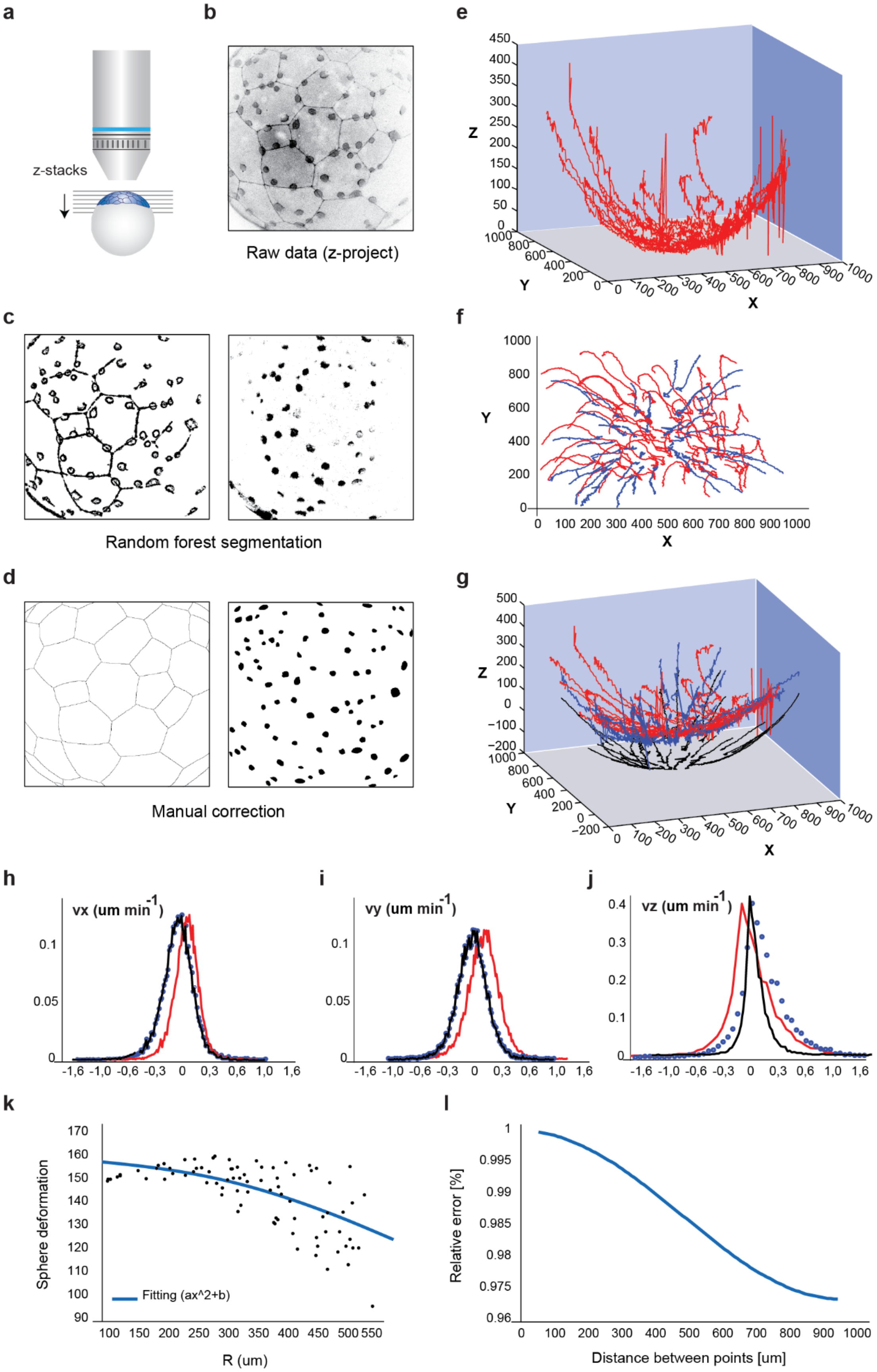
a
b
e
z-stacks
Raw data (z-project)
c
f
Random forest segmentation
d
g
Manual correction
h
i
j
vx (um min-1)
vy (um min-1)
vz (um min-1)
k
l
Sphere deformation
Fitting (ax^2+b)
R (um)
Relative error [%]
Distance between points [um]
X
Y
Z

Supplementary Figure 14 | Volume segmentation procedure and error estimation. (**a-d**) 2D segmentation. The x-y position of DCL cells and EVL vertices are extracted semi-automatically with a supervised classifier first followed by a manual check (see "Cell segmentation" section in Methods). In this stage, error is in the worst case of the size of one DCL cell (r=30 µm approximately). (**e-g**) Example of 3D processing used for tracking EVL cells. Raw 3D trajectories were approximated by locating the maximum z-intensity at each xyt location (red in **e**). A drift-correction algorithm was then applied removing drift artefacts in the trajectories (red), which after correction become radial, as expected (blue). Finally, the z coordinate is estimated by a projection onto a sphere (black trajectories) to remove noise, also deforming the egg surface (**g**). (**h-j**) Example of drift and sphere projection effects on DCL cell displacement. Histograms for vx (**h**) and vy (**i**) confirm that the drift correction does not change the distribution shape but does shift the curve, centring the histogram around 0, as expected, since DCL cells move away in all direction from the animal pole. After drift removal and sphere projection, the vz histogram (**j**) is not centred around 0 as expected, due to the collective movement from animal to vegetal (red: raw 3D; blue: after drift correction; black: after drift correction and sphere projection). (**k**,**l**) Example of sphere projection effects on DCL distances and error estimation. (**k**) Sphere projection effects as function of the egg radius for the last analysed frame of Supplementary Movie 1, where error decreases as moving away from the pole. (**l**) Error of measuring distances on the deformed surface relative to real z-axis distance. To provide an estimation of error due to this deformation, we focused on a basic function used extensively in the paper: the Euclidean 3D distance, specifically in z coordinate. Error is reported from the closest possible value (2 DCL cell radius), to a maximum distance between two points of $2^{1/2}$ R (R is the egg radius). To estimate the error we computed the ratio between the distance on the sphere vs the real distance where on the sphere distances were approximated using the fitting shown in panel k. The graph shows that the error ranges from around 0 % (for two DCL cells in contact) to 3 % (for very distant DCL cells).

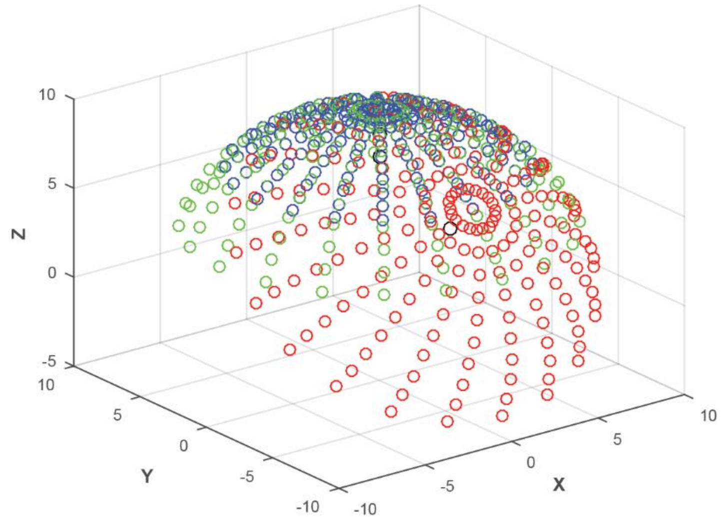

Supplementary Figure 15 | Drift correction. Blue circles represent the initial position of EVL cell vertices (*n*=201). Red circles represent the location of those points after isotropic growing on a sphere (R=10 um) and rotational drift (45 º). Green circles represent EVL vertex position after drift removal algorithm applied to red data sets and corrected to match blue positions set (error was 2.5 x 10^{-15} um, s.e. 5.3 x 10^{-17}).

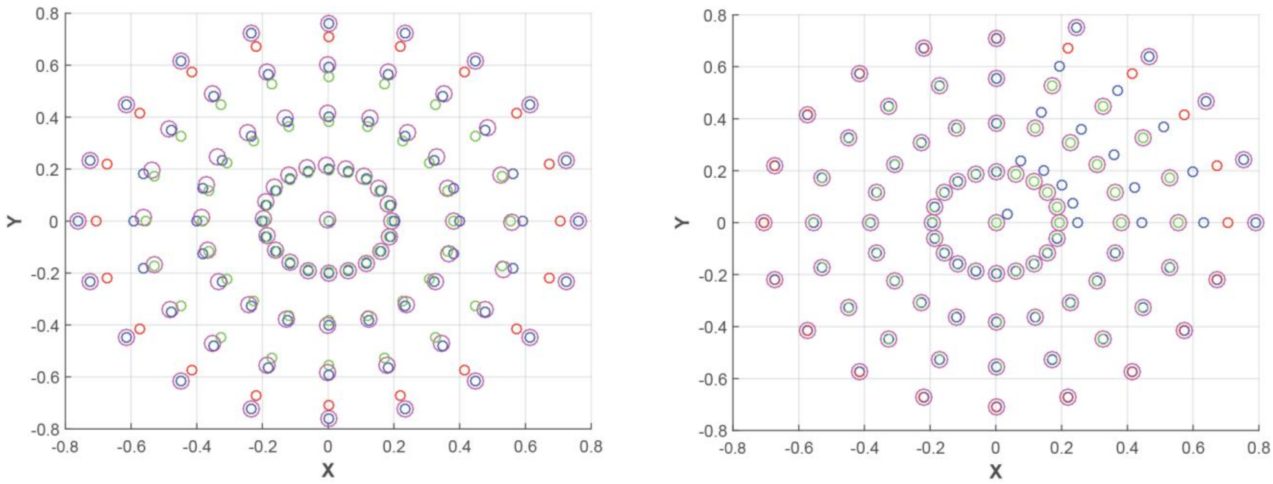

Supplementary Figure 16 | Independent motion estimation. Red and green circles represent the initial position of EVL cells for contour and non-contour positions, respectively; blue circles correspond to the final positions. For the algorithm estimations, only red positions are available to estimate EVL deformation (red/green to blue movement). Magenta circles are the estimation by the Delaunay based algorithm for isotropic (left) and anisotropic (right) deformations. Mean deformation error was 0.53M and 0.1M, where M is the maximum deformation in that scenario.

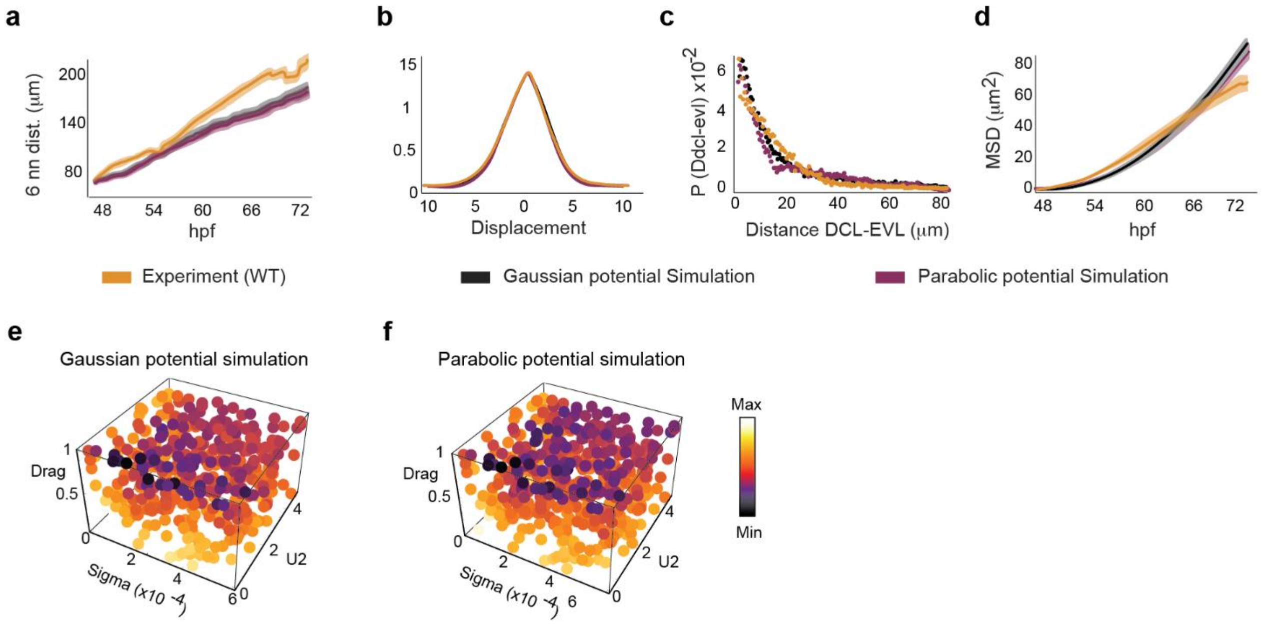

Supplementary Figure 17 | Comparison of the performance of simulations using gaussian and parabolic EVL cell border attraction potentials. (**a-d**) The performance of simulations considering gaussian and parabolic potentials of EVL cell border attraction are compared to experiments for the mean distance to the six nearest neighbours within the DCL (**a**), the probability density function of the displacement of DCL cells (**b**), the probability distribution of the distance of DCL cells to the nearest EVL cell border (**c**), and the mean square displacement (**d**). Gaussian and parabolic potentials give similar results for most figures of merit. However, the probability distribution of the distance of DCL cells to EVL cell borders obtained after a gaussian potential fits better the smooth increase towards shorter distances seen in the experiments (**c**). (**e-f**) Sampling of the free parameters D (drag), σ (sigma) and U_2. Colours indicate the normalised values of the target functions (according to the colour scale on the right) for different values of the free parameters. Gaussian and parabolic potentials give similar results but minimum values of drag, σ and U_2 concentrate in a smaller region for the gaussian potential.

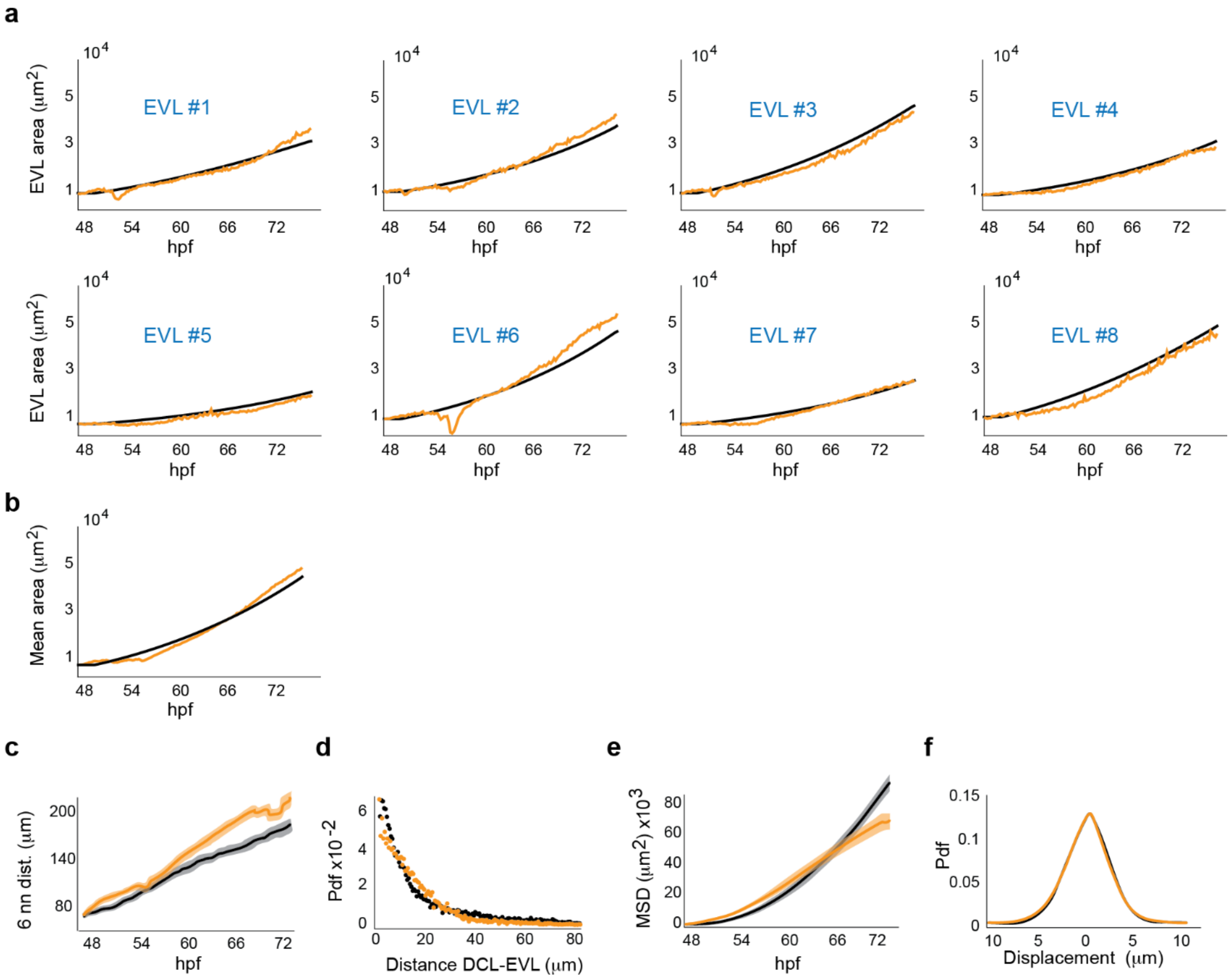

Supplementary Figure 18 | Comparison of target functions for EVL expansion and DCL spreading between experimental and simulation conditions. (**a**) Temporal changes in surface area of 8 individual EVL cells. (**b**) Temporal changes in the mean EVL cell surface area. (**c**) Temporal changes in the mean distance of a DCL cell to the six nearest neighbours (nn). (**d**) Probability distribution of the distance of DCL cells to the nearest EVL cell border. (**e**) Mean square displacement. (**e**) Probability density function of the displacement of DCL cells. Black and orange lines correspond to experiments and simulations, respectively.

Supplementary Tables

Supplementary Table 1 | Parameters of simulations

Symbol	Parameters	Free parameters	Value	Units
	**Egg**			
T_{exp}	Experiment duration		29	h
R_{egg}	Egg radius		593	μm
	**EVL**			
N_{evl}	EVL cell vertices number		133	
U_0	Spring constant EVL	*	26.79	h^{-1}
V_0	Velocity of the EVL margin		$0.03R_{egg}$	$\mu m\ h^{-1}$
	**DCL**			
N_{dcl}	Initial DCL number		80	
σ	Noise amplitude DCL	*	$0.0002\ R^2_{egg}$	$\mu m^2\ h^{-1}$
U_1	Magnitude repulsion potential DCL	*	$0.0003\ R^2_{egg}$	$\mu m^2\ h^{-1}$
a_1	Typical DCL radius		$0.036\ R_{egg}$	μm
	**EVL-DCL**			
D_r	Drag from EVL to DCL	*	1	
U_2	Magnitude of border pot. EVL-DCL	*	0.34	h^{-1}
a_2	Width border Gaussian pot. EVL-DCL		$0.03\ R_{egg}$	μm

Supplementary References

1. Castaneda, V. *et al.* Computational methods for analysis of dynamic events in cell migration. *Curr Mol Med* **14**, 291-307 (2014).
